# Hold your breath – Differential behavioral and sensory acuity of mosquitoes to acetone and carbon dioxide

**DOI:** 10.1371/journal.pone.0226815

**Published:** 2019-12-30

**Authors:** Majid Ghaninia, Shahid Majeed, Teun Dekker, Sharon R. Hill, Rickard Ignell

**Affiliations:** 1 School of Life Sciences, Arizona State University, Tempe, AZ, United States of America; 2 Division of Entomology, Department of Plant Protection, Gorgan University of Agricultural Sciences and Natural Resources, Gorgan, Iran; 3 Disease Vector Group, Unit of Chemical Ecology, Department of Plant Protection Biology, Swedish University of Agricultural Sciences, Alnarp, Sweden; University of Richmond, UNITED STATES

## Abstract

Host seeking in the yellow fever mosquito, *Aedes aegypti*, and the African malaria mosquito, *Anopheles coluzzii*, relies on specific and generic host-derived odorants. Previous analyses indicate that the behavioral response of these species depends differentially on the presence of carbon dioxide (CO_2_) and other constituents in human breath for activation and attraction. In this study, we use a flight tube assay and electrophysiological analysis to assess the role of acetone, a major component of exhaled human breath, in modulating the behavioral and sensory neuronal response of these mosquito species, in the presence and absence of CO_2_. When presented alone at ecologically relevant concentrations, acetone increases attraction in *Ae*. *aegypti*, but not in *An*. *coluzzii*. Moreover, in combination with CO_2_, human breath-equivalents of acetone ranging between 0.1 and 10 ppm reproduces a behavioral response similar to that observed to human breath in host-seeking *Ae*. *aegypti*, but not in *An*. *coluzzii*. Acetone does, however, reduce attraction to CO_2_ in *An*. *coluzzii*, when presented at a higher concentration of 10 ppm. We identify the capitate peg A neuron of the maxillary palp of both species as a dual detector of CO_2_ and acetone. The sensory response to acetone, or binary blends of acetone and CO_2_, reflects the observed behavioral output in both *Ae*. *aegypti* and *An*. *coluzzii*. We conclude that host recognition is contextual and dependent on a combination of ecologically relevant odorants at naturally occurring concentrations that are encoded, in this case, by differences in the temporal structure of the neuronal response. This information should be considered when designing synthetic blends for that optimally attract mosquitoes for monitoring and control.

## Introduction

The yellow fever mosquito, *Aedes aegypti*, and the African malaria mosquito, *Anopheles coluzzii* (formerly *Anopheles gambiae* molecular form M), exhibit a feeding preference for humans, making them efficient vectors of disease [1]. To identify new vector control measures to prevent the transmission of these diseases, the odor-mediated host-seeking behavior of mosquitoes has been put under scrutiny [2]. This behavior is heavily dependent on specific and generic host-derived volatile cues, which when presented in an accurate blend elicit a sequence of behaviors, including activation, attraction and landing [3–6]. Recognition of ´host´ differs across species, and is highly contextual and dependent on quantitative and qualitative differences in the odor blends [4, 5, 7]. Thus, an increased understanding of odor blend perception in mosquitoes is pertinent for the development of synthetic blends that support monitoring and control of host-seeking mosquitoes.

We, and others, have previously shown that sensory neurons in the maxillary palps of mosquitoes are tuned to constituents of vertebrate breath, including carbon dioxide (CO_2_) and (*R*)-1-octen-3-ol [4, 5, 8, 9], which are commonly used for the control and monitoring of mosquitoes. These studies show that interspecific variation in the behavioral response to volatile cues, when presented in ecologically relevant context and concentrations, can be explained by differential constraints on the olfactory system between mosquito species [4, 5]. To further analyze the behavioral and sensory response of *Ae*. *aegypti* and *An*. *coluzzii* to ecologically relevant odor blends, we here assess the role of another constituent of vertebrate breath, acetone, in the presence and absence of CO_2_.

Acetone is a by-product of fat metabolism and is a major component in exhaled breath, as well as in fresh urine and blood of humans and animals alike [10–14]. The concentration of acetone in human breath ranges from 0.5 to 2 ppm, but is greatly impacted by factors such as health, age and diet [12]. Similarly, acetone concentration in the breath of other animals, such as cattle, depends on the health of individual animals [10, 15, 16], and may reach concentrations of up to 40× higher than that of humans [11, 16].

Behavioral responses to acetone, either alone or in the presence of CO_2_, have been demonstrated for both *An*. *gambiae* [17, 18] and *Ae*. *aegypti* [19–21]. Besides enhancing the behavioral response to CO_2_, acetone, when presented at concentrations substantially higher than that found in human breath, affects the behavioral response to both complex host odors and individual constituents thereof [17, 19, 21, 22]. The behavioral response to acetone, however, differs among species. While acetone increases flight behavior in *An*. *gambiae*, it does not evoke landing and may even inhibit attraction to other host odorants [17, 19]. For *Ae*. *aegypti*, however, acetone, either alone or in combination with other host volatiles, increases attraction [20]. This suggests that acetone is differentially detected and encoded by the olfactory system of mosquitoes. As of yet, however, no studies have reported on which sensory neuron(s) in mosquitoes are responsible for the detection of acetone, or how this information is encoded.

In this study, we analyze the behavioral response of female *Ae*. *aegypti* and *An*. *coluzzii* to human breath and binary blends of acetone and CO_2_, as well as to acetone and CO_2_ alone, at ecologically relevant concentrations [5, 10–12, 15, 16, 23–25]. Physiological responses to these stimuli by the CO_2_-sensitive neuron on the maxillary palp of the two species reflect the species-specific differences in behavioral responses. We discuss our findings in relation to previous studies and place them in the context of development of attractants for disease-transmitting mosquitoes.

## Materials and methods

### Mosquitoes

*Aedes aegypti* (Rockefeller strain) and *An*. *coluzzii* (Suakoko strain; previously *An*. *gambiae* molecular form M) were reared at 27 ± 2°C, 70 ± 2% relative humidity (RH) under a 12 h : 12 h light : dark period, as previously described [26]. For all experiments, 6-day post-emergence female mosquitoes were used; these were provided water but deprived of their regular sugar source 24 h prior to experiments.

### Flight tube bioassay

**Fig 1:**
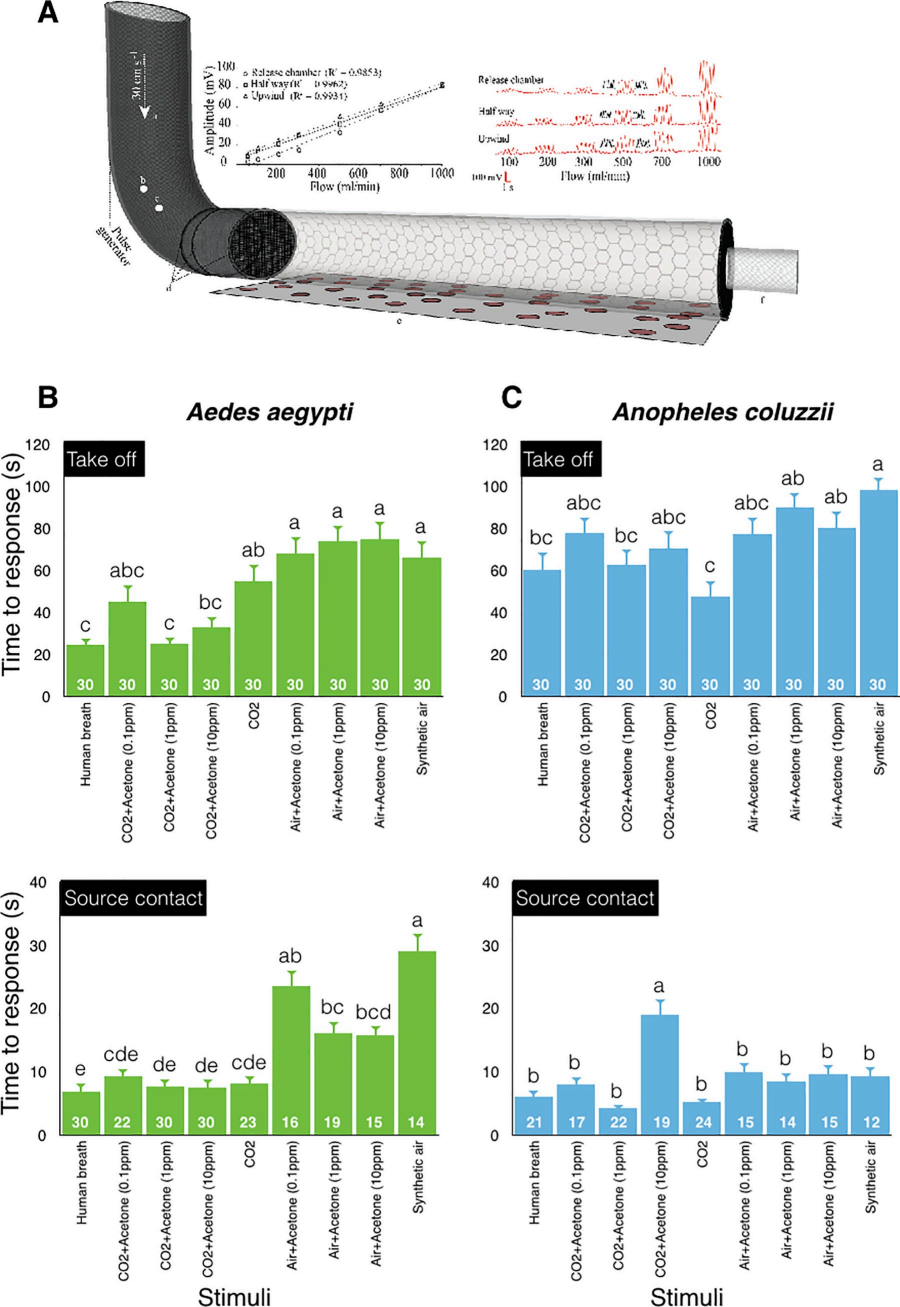
The behavioral response of host-seeking *Aedes aegypti* and *Anopheles coluzzii* to synthetic air, human breath, acetone and CO_2_, as well as binary blends thereof. **A.** Behavior was assessed in a flight tube assay: (a) charcoal filtered and humidified air flowed through the assay, (b) pressurized air inlet, (c) stimulus inlet into which the stimulus was injected, (d) stainless-steel mesh plume diffusers, (e) glass flight tube, and (f) release chamber. The upper panels demonstrate that the pulsed stimuli (here shown as five cycles of 1 s on and 1 s off) maintain their amplitude and shape throughout the flight tube and at all tested flow rates. The upper right panel shows the consistent and distinct pulsed stimuli at ascending flow rates of known concentration of acetone in the flight tube. Discrete pulsed stimuli were measured in the center (in red) and at the lateral sides (in black) of the release chamber, at halfway and at the source. The upper left panel presents a graphical representation of the distinct pulsed stimuli, which shows the average amplitude of each of the five distinct pulses (N = 10) at different positions and the regression correlation coefficients (R^2^) that demonstrate the consistency of the stimulus amplitude at the different positions within the flight tube with increasing flow rates. Time to take off (**B**, **C**; top graphs) and source contact (**B**, **C**; bottom graphs) are differentially affected by human breath, acetone and by the binary blends in the two species. The number of mosquitoes responding is indicated as numbers inset in each bar. Different letters above the bars denote significant differences between treatments within species (ANOVA, p < 0.05). Vertical bars represent the standard error of means ± SE.

To analyze the behavioral response of *Ae*. *aegypti* and *An*. *coluzzii* to exhaled breath, as well as to acetone and CO_2_, presented alone or in binary blends, a flight tube bioassay was used (Fig 1A; [5]. The bioassay was illuminated from above with white light at 280 lux for the diurnal *Ae*. *aegypti*, while red light (40 lux) was used for the nocturnal *An*. *coluzzii*. Experiments were conducted during their period of peak host-seeking activity of each species [27, 28]. Charcoal-filtered humidified air (25 ± 2°C, RH 65 ± 2%) flowed through the flight tube at 30 cm s^−1^. To ensure a laminar flow and a homogeneous plume structure, the air passed through a series of stainless-steel mesh screens prior to entering the flight tube. Homogenous discrete stimulus pulses (1 s on and 1 s off) were introduced into the flight section using a stimulus controller (SEC-2/b, Syntech), which pumped stimulus-laden air from a 40 l Tedler gas sampling bag (Adtech Polymer Ltd, Stroud, UK) into a pulse generator, placed behind the mesh screens [24]. Stimuli were prepared in the following way: 1) breath was collected by a volunteer exhaling normally through a mouthpiece into the bag for 4 min prior to the experiment; 2) 30000 ppm CO_2_ was prepared by introducing synthetic air (Strandmöllen AB, Ljungby, Sweden) at 1.5 l min^-1^ and pure CO_2_ (Strandmöllen AB) at 0.2 l min^-1^ into the gas sampling bag, to obtain the concentration approximating the natural composition in exhaled breath [12]; 3) acetone (99.9%, Chromasolv, Sigma-Aldrich, Stockholm, Sweden) was diluted with distilled water and then pipetted into the bag, which was subsequently filled with synthetic air, to obtain concentrations of 0.1 ppm, 1 ppm and 10 ppm at the downwind end of the flight tube; 4) binary mixtures of 30000 ppm CO_2_ and the three concentrations of acetone; and 5) for the negative control experiments, the gas sampling bag was filled with synthetic air at an airflow of l.5 l min^-1^. The consistency in amplitude and the structure of the discrete pulsed stimuli was visualized using a mini-PID (Aurora Scientific, Aurora, Ontario, Canada) with known concentration of acetone with different flow rates as described by Majeed et al. [24]. In addition, the concentrations of acetone and CO_2_ were measured at the down-wind end of the flight tube using the mini-PID (Aurora Scientific, Aurora, Ontario, Canada) and a CO_2_ analyzer (LI-820, LICOR Biosciences, Lincoln, NE, USA), respectively. This revealed a concentration of 1200 ± 12 ppm CO_2_, as well as average concentrations of 0.1 ± 0.02 ppm, 1 ppm ± 0.01 and 10 ± 0.04 ppm acetone.

Individual mosquitoes were kept in glass release chambers (7 × 2.6 cm i.d.), covered with stainless steel mesh on one side and a cotton plug on the other, in the bioassay room for 24 h prior to the experiments [5]. Following the opening of the release chamber, the time to take off (flight activation) and the time to source contact were recorded. The maximum experimental time was 120 s. Thirty individuals of each species were observed for all treatments. To minimize the effect of daily variation in baseline activity and responses to odors, an equal number of test and control individuals were observed each day. For both species, to compare the time to take off and the time to source contact in response to the various treatments (human breath, CO_2_ and acetone alone, binary blends and synthetic air), an ANOVA followed by Tukey’s HSD *post hoc* test was used (GraphPad Prism, v. 7; GraphPad software, La Jolla, CA, USA). The total number of mosquitoes making source contact was analyzed with nominal logistic regression, comparing presence and absence of CO_2_ and dose of acetone for each species (JMP Pro v. 14, SAS Institute Inc., Cary, NC, 1989–2019).

### Electrophysiology

Initial screening of all previously characterized functional types of sensilla on the antennae of *Ae*. *aegypti* and *An*. *coluzzii* [26, 29–32] revealed that the sensory neurons sensitive to acetone are found on the maxillary palps. Specifically, the type of sensory neuron responding to acetone is the same as the one responding to CO_2_, the capitate peg A (cpA) neuron [5]. Electrophysiological recordings from this sensillum type were made and analyzed as previously described [5].

A continuous humidified stream of synthetic air (Strandmöllen AB), lacking CO_2_, was passed over the maxillary palp (2 l min^−1^) via a glass tube (7 mm i.d.). Stimuli, consisting of CO_2_, acetone or binary mixture thereof, were introduced into the air stream through a hole (2 mm i.d.) in the glass tube, 11 cm upstream of the maxillary palps. Delivery of CO_2_ was regulated by a two-way Teflon solenoid valve (Teddington, Skogås, Sweden), controlled via the digital output of an IDAC-4 (Syntech, Germany). The valve was connected to a separate gas cylinder containing 1200 ppm CO_2_ and oxygen (20%), balanced by nitrogen (Strandmöllen AB). Acetone, dissolved in distilled water, at concentrations ranging from 0.1 to 100000 ppm, were loaded (15 μl) onto a piece of filter paper (5 × 20 mm), placed inside a Pasteur pipette. The pipettes were sealed using Parafilm^™^ after loading and used once within 5 min to limit the variation due to evaporation. For the dose-response analysis, stimuli were presented in increasing concentrations from 0.1 to 100000 ppm. To test binary mixtures of CO_2_ and acetone, pipettes loaded with 0.1, 1 and 10 ppm acetone were gently, yet rapidly, filled with 1200 ppm CO_2_, after establishing a stable contact with a capitate peg sensillum. The rationale for selecting 1200 ppm CO_2_ for the binary blends was that this concentration corresponds with the natural level associated with proximity to a host [23, 25] and has previously been shown to elicit behavioral response in both mosquito species [5, 24]. Stimuli were presented in the following order: CO_2_ (1200 ppm), 0.1 ppm acetone, 0.1 ppm acetone + CO_2_, 1 ppm acetone, 1 ppm acetone + CO_2_, 10 ppm acetone, 10 ppm acetone + CO_2_. For all experiments, distilled water was used as a control. The entire panel of stimuli was used once per specimen. The time between stimuli was 10 s. Extracellular spike activity of the A neuron was analyzed by counting the number of spikes 0.5 s before stimulus onset and subtracting this from that of the 0.5 s period following stimulus delivery. Results are presented as spikes s^-1^. To further analyze the neural response to the binary mixtures, the temporal characteristics were analyzed by plotting frequency histograms (spikes s^-1^) during 100 ms bins over a period of 2s (500 ms before to 1500 ms after the response onset).

To compare the sensitivity of the capitate peg A neurons of the two species to acetone and the binary blends, a repeated measures two-way ANOVA followed by Tukey’s multiple comparison test was used (GraphPad Prism, v. 8.2.1). The kinetic curve fits were generated using a non-linear regression association/dissociation model (association then dissociation model constrained at HotNM = 12 and Time_0_ = 0.2; GraphPad Prism, www.graphpad.com/guides/prism/8/curve-fitting/reg_equaton_association_then_disso.htm).

## Results

### Reliance on acetone for the behavioral response to human breath

The behavioral response of *Ae*. *aegypti* and *An*. *coluzzii* was differentially affected by acetone and the binary blends (Take off: F_(8,261)_ = 9.93, P < 0.0001 and F_(8,261)_ = 4.43, P < 0.0001, respectively; Source contact: F_(8,190)_ = 15.2, P < 0.0001 and F_(8,150)_ = 7.22, P < 0.0001, respectively) (Fig 1). Compared to synthetic air, acetone alone did not affect the time to take off in either species, but significantly reduced the time to source contact in *Ae*. *aegypti* (Fig 1B and 1C). When presented together with CO_2_, on the other hand, acetone significantly reduced the time to take off in *Ae*. *aegypti*, but not in *An*. *coluzzii* (Fig 1B and 1C; top). Combinations of acetone and CO_2_ did not influence the time to source contact in *Ae*. *aegypti* (Fig 1B; bottom), but the time to source contact increased at the highest dose tested in *An*. *coluzzii* (Fig 1C; bottom). For both species, the number of insects that made source contact was higher in the presence of CO_2_ but was not affected by the dose of acetone (*Ae*. *aegypti* χ^2^ = 1.32, p = 0.52, parameter estimates: intercept = 0.036, acetone = 0.63; *An*. *coluzzii* χ^2^ = 1.51, p = 0.93, parameter estimates: intercept = 0.031, acetone = 0.46).

### Detection of acetone

**Fig 2:**
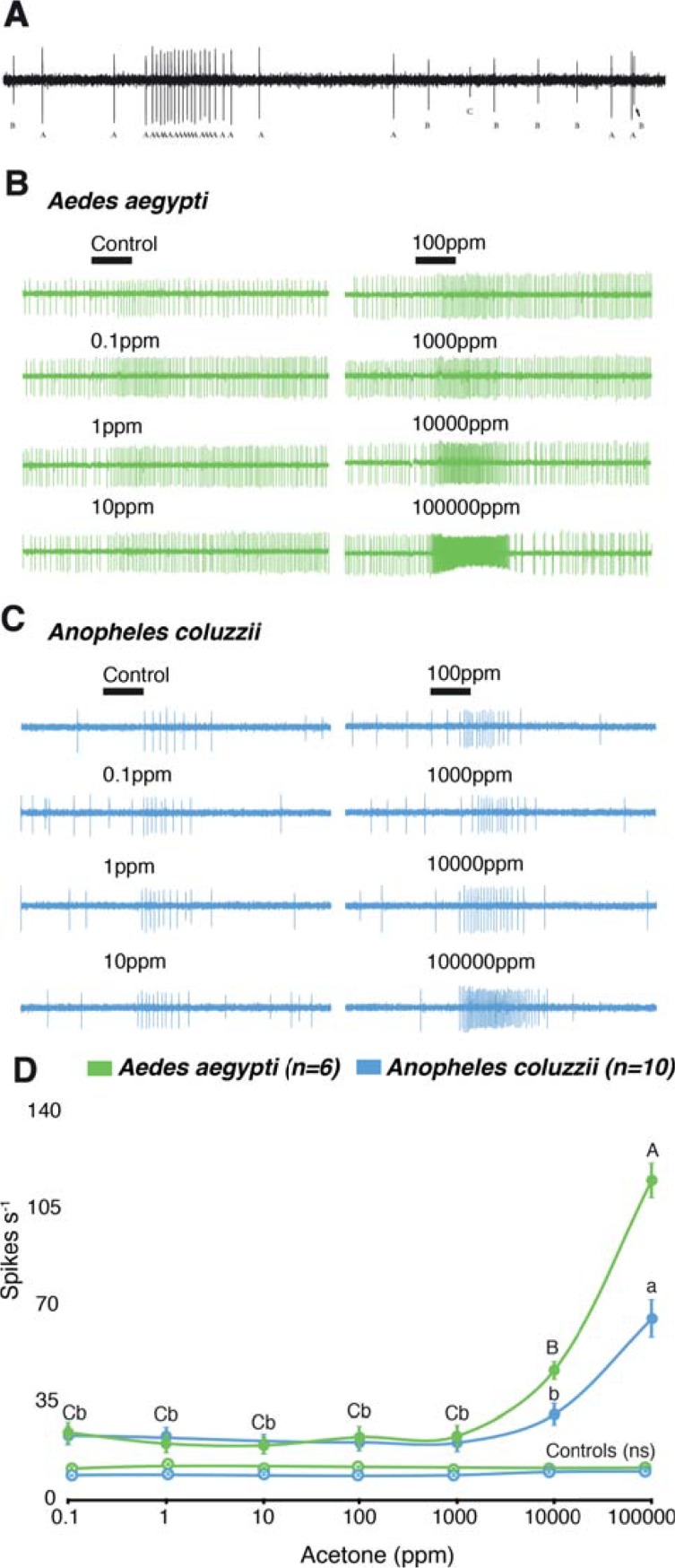
Response of the capitate peg A (cpA) neuron of *Aedes aegypti* and *Anopheles coluzzii* to acetone. **A**. Sample trace from a capitate peg sensillum of *Ae*. *aegypti*, displaying the firing frequency of the three neurons (cpA, B, and C). Dose-dependent response of the cpA neuron in *Ae*. *aegypti* and *An*. *coluzzii* to increasing concentrations of acetone (**B-D**). Note the significant increased response, above the control, to low concentrations of acetone (**D**). At higher concentrations, acetone elicits a significantly higher response in the cpA neuron of *Ae*. *aegypti* than of *An*. *coluzzii*, indicated by different letter designations (two-way repeated-measures ANOVA, Tukey’s multiple comparison test, uppercase, *Ae*. *aegypti*; lowercase, *An*. *coluzzii*) (**D**). “ns” indicates no significant difference among the doses for each species control.

Electrophysiological recordings from the capitate peg sensilla of *Ae*. *aegypti* and *An*. *coluzzii* (Fig 2A) revealed a dose-dependent response of the overall rate of firing of the cpA neuron to acetone at concentrations exceeding 1000 ppm (Dose: F_(6, 196)_ = 62.34, p < 0.0001), with *Ae*. *aegypti* being significantly more sensitive than *An*. *coluzzii* (Species: F_(3, 196)_ = 121.3, p < 0.0001) (Fig 2B–2D). Interestingly, even at low, and ecologically relevant, concentrations (0.1–100 ppm), acetone elicited a higher response than the control (synthetic air) for both *Ae*. *aegypti* (p = 0.0097) and *An*. *coluzzii* (p = 0.0047) (Fig 2B–2D). Taken together, these results demonstrate that both species are capable of sensing low, ecologically relevant, concentrations of acetone, however assessing the effect of the dose requires higher resolution analysis.

### Detection of binary blends of acetone and CO_2_

**Fig 3:**
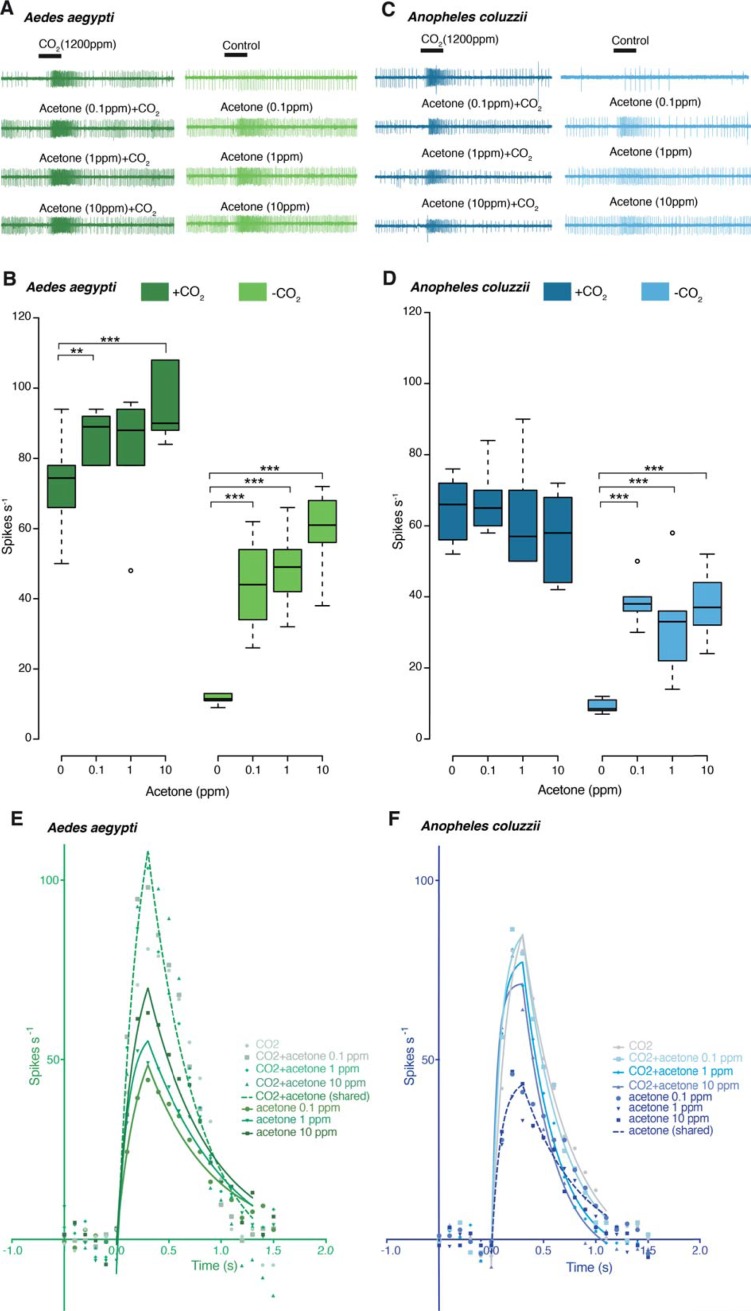
Electrophysiological response of the capitate peg A (cpA) neuron of *Aedes aegypti* and *Anopheles coluzzii* to acetone, CO_2_, and binary blends thereof. Physiological response of the cpA neuron in *Ae*. *aegypti* (**A, B**) and *An*. *coluzzii* (**C, D**) to increasing concentrations of acetone, CO_2_, and binary blends thereof. **E** and **F** show, in high temporal resolution, the differential increase in response of the cpA neuron of *Ae*. *aegypti* (n_recording_ = 6, N_individual_ = 6) and *An*. *coluzzii* (n_recording_ = 6, N_individual_ = 6) to acetone in the presence or absence of CO_2_ (two-way repeated-measures ANOVA, Tukey’s multiple comparison test; ** p < 0.01; *** p < 0.001).

The response of the cpA neuron, when analyzed at high temporal resolution, to either acetone or CO_2_ alone, was significantly different from that to the binary blends for both *Ae*. *aegypti* (F_(1,10)_ = 70.84, p < 0.0001) and *An*. *coluzzii* (F_(1,10)_ = 53.04, p < 0.0001) (Fig 3A–3D). In *Ae*. *aegypti*, the cpA response to acetone was significantly higher than that to the control, in both the presence and absence of CO_2_ (F_(3,30)_ = 30.04, p < 0.0001) (Fig 3E; Table 1). In contrast, in *An*. *coluzzii*, the only significant change in cpA activity was to acetone in the absence of CO_2_ (F_(3,30)_ = 6.47, p = 0.0017) (Fig 3F; Table 1).

**Table 1:** The goodness of fit for the non-linear regressions in Fig 3C and 3D.

		CO_2_	CO_2_+ acetone 0.1 ppm	CO_2_+ acetone 1 ppm	CO_2_+ acetone 10 ppm	CO_2_+ acetone (shared)	acetone 0.1 ppm	acetone 1 ppm	acetone 10 ppm	acetone (shared)
*Ae*. *aegypti*	Degrees of Freedom	66	80	80	80	318	80	80	80	248^a^
	R^2^	0.70	0.86	0.72	0.74	0.74	0.56	0.71	0.66	0.61^a^
	Adjusted R^2^	0.69	0.85	0.71	0.73	0.74	0.54	0.69	0.64	0.60^a^
	Sum of Squares	241.00	163.40	347.00	458.50	1282.00	119.30	103.40	174.10	457.10^a^
	Sy.x	1.91	1.43	2.08	2.39	2.01	1.22	1.14	1.48	1.36^a^
*An*. *coluzzii*	Degrees of Freedom	68	68	68	68	284^a^	68	68	68	212
	R^2^	0.80	0.84	0.78	0.73	0.76^a^	0.72	0.51	0.61	0.59
	Adjusted R^2^	0.79	0.83	0.77	0.72	0.76^a^	0.70	0.49	0.59	0.59
	Sum of Squares	136.60	111.40	160.00	204.20	679.10^a^	53.69	108.70	109.00	282.00
	Sy.x	1.41	1.28	1.53	1.73	1.55^a^	0.89	1.26	1.27	1.15

^a^Model rejected by extra sum of squares F test at 𝛼 = 0.05.

The temporal pattern of response to acetone and CO_2_ alone, as well as binary blends thereof, was phasic-tonic for both *Ae*. *aegypti* and *An*. *coluzzii* (Fig 2B and 2C and Fig 3C, A,C In *Ae*. *aegypti*, the cpA activity in response to CO_2_ and the three binary blends of acetone and CO_2_ could be modeled by a single, shared association/dissociation curve (F_(12,306)_ = 1.509, p = 0.1194; dotted line in Fig 3E), revealing a consistently higher kinetic response to increasing concentrations of acetone in combination with CO_2_ compared to acetone alone (Fig 3E). In contrast, a model of the activity of the *Ae*. *aegypti* cpA neuron to acetone alone revealed that each concentration could be represented by distinct association/dissociation curves (F_(8,240)_ = 4.551, p < 0.0001; solid lines in Fig 3E), demonstrating that the response kinetics of this neuron is influenced by acetone concentration (Fig 3E). The reverse was observed for *An*. *coluzzii*, in which the cpA response to acetone alone could be modeled by a single, shared curve (F_(2,204)_ = 1.566, p = 0.1367; dotted line in Fig 3F), while the response to CO_2_ and the blends were modeled individually (F_(12,272)_ = 4.198, p < 0.0001; solid lines in Fig 3F). This demonstrates that the response kinetics of this neuron is influenced by acetone concentration only in the presence of CO_2_ (Fig 3F).

## Discussion

The behavioral response of *Ae*. *aegypti* and *An*. *coluzzii* to human breath relies on the presence of CO_2_ [18, 33, this study] and is differentially modulated by acetone. While acetone enhances the behavioral response of *Ae*. *aegypti*, in both the presence and absence of CO_2_, it decreases the behavioral response of *An*. *coluzzii* in the presence of CO_2_. This demonstrates that blend perception of generic volatile compounds in human breath plays a role in host attraction for these species. The observed differences in behavioral attraction correlate with the different mode of encoding CO_2_ and acetone, as well as the binary blends, by the cpA neurons of the two species. The data provided here, together with our previous studies [4, 5], clearly emphasize that generic host volatiles, when presented in ecologically relevant concentrations, may provide reliable cues for host attraction and recognition for mosquitoes.

When presented at ecologically relevant concentrations, acetone, in combination with CO_2_, reproduces a behavioral response similar to that observed to human breath of host-seeking *Ae*. *aegypti*, but not of *An*. *coluzzii*. This is in line with previous observations demonstrating that activation of host-seeking *Ae*. *aegypti* and *An*. *gambiae* differentially relies on human breath constituents other than CO_2_ [18, 33]. Acetone, when presented alone at concentrations of 1–10 ppm, significantly decreased the time to source contact in *Ae*. *aegypti* when compared with synthetic air. A similar observation was made by Venkatesh and Sen [20], albeit at concentrations of acetone >100,000× higher than that used in the current study, demonstrating that acetone may substitute for CO_2_, as suggested by Bernier et al. [21]. A plausible explanation for the observed behaviour at these extreme concentrations may be a result of the intense activation of the CO_2_ sensitive, cpA, neurons, demonstrated here. When presented in combination with CO_2_, 1 ppm acetone, an equivalent concentration of that found in human breath (0.5–2 ppm), significantly decreased the time to take off in *Ae*. *aegypti* compared to that of CO_2_ alone, to a level similar to that observed to human breath. Acetone has previously been shown to enhance the behavioral response of *Ae*. *aegypti* to other host volatiles, in the presence of CO_2_ [21, 22], verifying that acetone may act additively or synergistically to enhance activation and attraction. In contrast, acetone, whether in the presence or absence of CO_2_, did not significantly affect time to take off and source contact for *An*. *coluzzii*. The only exception to this was an observed increase in time to source contact, when acetone was presented at 10 ppm together with CO_2_. A similar inhibition of the behavioural response to high concentrations of acetone has been reported by Takken et al. [19] and Qiu et al. [17]. We conclude that *Ae*. *aegypti* and *An*. *coluzzii* appear differentially constrained in their capacity to respond behaviorally to binary blends of acetone and CO_2_.

The observed differential behavioral response of *Ae*. *aegypti* and *An*. *coluzzii* to acetone and CO_2_, and binary blends thereof, is reflected in the sensory response of the cpA neurons to these stimuli. While we cannot rule out the existence of rare types of acetone-sensitive neurons on the antennae or differences in higher order processing of either species, this study suggests that the CO_2_-sensitive neuron in mosquitoes, far from being a labelled line for CO_2_, is capable of encoding breath-related blends. While acetone is a novel ligand for the cpA neuron, other odorants are known to be agonists of the cpA neuron in *Ae*. *aegypti*, *An*. *gambiae* and in the southern house mosquito, *Culex quinquefasciatus*, when presented at high concentrations [34–36]. Our physiological analysis, however, reveals that the cpA neuron of both *Ae*. *aegypti* and *An*. *coluzzii* demonstrate a high sensitivity to ecologically relevant concentrations of acetone alone, which in *Ae*. *aegypti* correlates with behavior. A correlation is also found between the behavior of both *Ae*. *aegypti* and *An*. *coluzzii* and the sensory response of their cpA neurons to the binary blends. In *Ae*. *aegypti*, stimulation with both acetone and CO_2_ significantly increased the response of the cpA neuron, without altering the response kinetics, whereas the opposite was found for *An*. *coluzzii*. The observed differences in sensory response of the two species to acetone, either alone or in combination with CO_2_, is intriguing. Agonists of the CO_2_-sensitive neuron in both mosquitoes [34–36] and *Drosophila melanogaster* [35, 37] have been shown to be detected by gustatory receptors (Grs) expressed in the cpA neuron. Whether structural or stoichiometric differences of the Grs in the two mosquito species [8, 38, 39] account for the demonstrated differences in sensory response remain to be analyzed.

Previous studies indicate that behavioral responses to complex host odors in mosquitoes are more robust than to single host volatiles [3, 6, 17, 40, 41]. While host discrimination and selection by anthropophilic mosquitoes may be regulated by species-specific host volatiles [42, 43], recent research suggests that these behaviors also rely on a number of generic host volatiles and their relative proportions [3, 4, 41, 44–48]. In most of these studies, these host volatiles have been shown to be detected by sensory neurons on the antennae, and readily synergize with CO_2_ in eliciting host-related behaviors. In this study, we identify acetone as a host volatile, detected by sensory neurons on the maxillary palps, that differentially modulates both the physiological and behavioral responses in *Ae*. *aegypti* and *An*. *coluzzii*. Behaviorally, *Ae*. *aegypti* is activated by the presence of CO_2_ and acetone at ecologically relevant concentrations, whereas the activation of *An*. *coluzzii* is independent of the presence of acetone. This is reflected in the firing rates of the cpA sensory neuron of each species. In contrast, source contact is mediated in *Ae*. *aegypti* by CO_2_, with or without acetone, while this is not the case in *An*. *coluzzii*. Moreover, *An*. *coluzzii* tolerates only a limited concentration of acetone, less than 10 ppm, above which the time to source contract increases. This differential response to the presence of acetone correlates with the change in temporal kinetics seen in the cpA neuron in *An*. *coluzzii*. This suggests both that other stimuli are involved in attraction to the host in *An*. *coluzzii* [18], and that acetone is acting as a host discrimination cue in this species, but not in *Ae*. *aegypti*. In *Ae*. *aegypti*, acetone, together with CO_2_, is acting as an activator, similar to human breath, and may be the missing component in human breath that was proposed by Khan and Maibach [33] to be responsible for the observed greater human attraction.

While the role of CO_2_ in activating, sensitizing and attracting mosquitoes to potential hosts is well characterized [3, 5, 49, and references therein], this study reveals that another major component of exhaled breath, acetone, is able to modulate this behavioral response. When presented in binary blends together with CO_2_, acetone, within the natural concentrations found in the exhaled breath of potential hosts, enhances both the sensory and behavioral response of *Ae*. *aegypti*. For *An*. *coluzzii*, which is highly anthropophilic, the sensory and behavioral response is decreased at higher concentrations of acetone, indicating that acetone may act as a recognition cue to discriminate among hosts. From a vector control perspective, additional identification of ecologically relevant odorants and their naturally occurring concentrations, and how these factors affect vector-host interaction, can aid in optimizing synthetic blends for monitoring and control of mosquito populations.
